# Aetiology of nutritional rickets in rural Bangladeshi children

**DOI:** 10.1016/j.bone.2020.115357

**Published:** 2020-07

**Authors:** Sonia Ahmed, Gail R. Goldberg, Rubhana Raqib, Swapan Kumar Roy, Shahidul Haque, Vickie S. Braithwaite, John M. Pettifor, Ann Prentice

**Affiliations:** a MRC Human Nutrition Research, Elsie Widdowson Laboratory, Cambridge, UK; b International Centre for Diarrhoeal Disease Research, Bangladesh (ICDDR,B), Dhaka 1000, Bangladesh; c Social Assistance and Rehabilitation for the Physically Vulnerable (SARPV), Dhaka 1207, Bangladesh; d MRC Epidemiology Unit, University of Cambridge School of Clinical Medicine, Cambridge CB2 0SL, UK; e SAMRC/Wits Developmental Pathways for Health Research Unit, Department of Paediatrics, University of the Witwatersrand, Johannesburg, South Africa

**Keywords:** Bangladesh, Calcium, Fibroblast growth factor 23, Phosphate, Rickets, Vitamin D

## Abstract

**Objectives:**

A high prevalence of rickets of unknown aetiology has been reported in Chakaria, Bangladesh. Classically, rickets is caused by vitamin D deficiency but increasing evidence from Africa and Asia points towards other nutritional deficiencies or excessive exposure to some metals. The aim of this study was to investigate the aetiology of rickets in rural Bangladeshi children.

**Methods:**

64 cases with rickets-like deformities were recruited at first presentation together with age-sex-village matched controls. Data and sample acquisition included anthropometry, radiographs, fasted plasma and urinary samples, 24 h weighed dietary intake together with a 24 h urine collection, and ^13^C-breath tests to detect *Helicobacter (H.) pylori* infection.

**Results:**

One child had active rickets and frank hypovitaminosis D (F, *n* = 1) and one had deformities with radiological features of Blount disease (M, *n* = 1). The remaining cases were grouped into those with active rickets, defined as a radiographic Thacher score ≥1.5 (Group A, *n* = 24, 12M, 12F) and rickets-like bone deformities but not active rickets (Group B, *n* = 38, 28M, 10F). All children had a low dietary calcium intake, but this was lower in Group A than their controls (mean (SD): 156 (80) versus 323 (249) mg/day, *p* = 0.005). Plasma 25-hydroxyvitamin D (25OHD) was lower in Group A compared to controls; 63% of Group A and 8% of controls had a concentration <25 nmol/L (*p* ≤ 0.0001). There was, however, no evidence of differences in skin sunshine exposure. Group A had lower plasma calcium and phosphate and higher 1,25-dihydroxyvitamin D (1,25(OH)_2_D) and parathyroid hormone (PTH). 88% of Group A and 0% of controls had undetectable plasma intact fibroblast growth factor (iFGF23), with c-terminal FGF23 (cFGF23) concentrations in the normal range. Urinary phosphate and daily outputs of environmental metals relative to creatinine were higher and tubular maximal phosphate reabsorption per unit glomerular filtration rate (TmP/GFR) was lower in Group A compared to controls. Although less pronounced than Group A, Group B had higher alkaline phosphatase, 1,25(OH)_2_D and PTH concentrations than controls but similar calcium intake, TmP/GFR, iFGF23 and cFGF23 concentrations. Mean 25OHD concentrations were also similar to controls and there was no significant difference in the percentage <25 nmol/L (Group B: 13%, controls: 5%, *p* = 0.2) No group differences were seen in prevalence of anaemia, iron deficiency or *H. pylori* infection.

**Conclusion:**

Nutritional rickets in this region is likely to be predominantly due to low calcium intake in the context of poor vitamin D status and exposure to environmental metals, but not *H. pylori* infection, anaemia or iron deficiency.

## Highlights

• Rickets in Chakaria, Bangladesh was associated with low dietary calcium intake.
• Low vitamin D status (>clinical deficiency range) and exposure to metals were noted.
• Chronically low calcium intakes caused urinary phosphate wasting and rickets.
• Phosphaturia was not driven by elevated FGF23 rather secondary hyperparathyroidism.
• Rickets showed no relationship with *H. pylori* infection, anaemia or iron deficiency.

## 1 Introduction

Rickets is a major public health problem in Bangladesh affecting around 50,000 children in Chakaria in the south east region of the country alone. Previous studies have investigated vitamin D [1], calcium deficiency [2] or metal toxicity [3] as potential aetiological factors. Limitations of these studies include the lack of appropriate “healthy” local control groups, radiographic diagnosis of rickets, and standardised biochemical assays/techniques.

Children with rickets in Bangladesh tend to present after 2 years of age rather than in infancy [1,3,4]. This is of interest as the literature suggests that vitamin D deficiency rickets typically affects infants [1] and adolescents [5] during rapid periods of growth whereas studies of calcium-deficiency rickets in Africa suggest a presentation in childhood rather than infancy [6].

Studies from The Gambia [7,8], Malawi [9] and Nigeria [10] have pointed towards a chronically low dietary calcium intake leading to increased parathyroid hormone (PTH) and 1,25-dihydroxyvitamin D (1,25(OH)_2_D) concentrations and lower plasma phosphate concentration coupled with higher urinary phosphate loss compared with controls. In the main, children from these studies had 25-hydroxyvitamin D (25OHD) concentrations >25 nmol/L thus reducing the likelihood of vitamin D deficiency as the primary aetiological factor [6]. Nevertheless, 25OHD concentration tended to be lower in children with rickets compared to controls. A Gambian study found that children with previous rickets had a faster turnover of isotopically-labelled 25OHD compared with controls [11] which suggests a greater requirement for vitamin D in the presence of calcium deficiency. In addition, studies from The Gambia have found plasma c-terminal fibroblast growth factor-23 (cFGF23) concentration to be greatly elevated and for iron deficiency to be more prevalent in children with rickets-like bone deformities compared with controls suggesting a potential role for iron deficiency in FGF23 metabolism and rickets aetiology [12].

The aim of this study was to perform a comprehensive case-control study of newly, radiographically-diagnosed children with rickets and age-, sex- and village- matched controls in Chakaria, Bangladesh paying particular attention to calcium, vitamin D, phosphorus and iron metabolism, dietary intake, and exposure to metals and infections which may interfere with intestinal calcium absorption. The main hypothesis was that urinary phosphate wasting as a result of elevated FGF23 production caused by calcium deficiency is the underlying mechanism leading to rickets in children in Chakaria.

## 2 Materials and methods

### 2.1 Recruitment and criteria for inclusion and exclusion

The study was approved by the Research and Ethics Review Committees of International Centre for Diarrhoeal Disease and Research, Bangladesh (ICDDR,B). Participants were recruited from the villages of Cox's Bazar subdistrict of Chakaria in the south-eastern region of Bangladesh (21°N), which had the highest prevalence of rickets in Bangladesh (2.1%) [13]. Children showing signs of rickets, which had not previously been diagnosed or treated, presented or were referred to the SARPV (Social Assistance and Rehabilitation for the Physically Vulnerable, http://sarpv.org) clinic in the district where a more detailed examination was performed and radiographs of wrists and knees taken. Diagnosis of rickets was made according to SARPV criteria based on the presence of both clinical and radiographic signs of rickets. Following the Thacher Scoring system, a score was assigned to each potential rickets case [14]. If the total score was ≥1.5, the patient was diagnosed as having radiologically active rickets. However, SARPV radiographic scoring method included only the radius and femur but not smaller bones ulna, tibia and fibula (so in total score = 5*2 = 10). Therefore, it was decided that, after completion of fieldwork, a second independent opinion from one of the authors (JP) would be sought to confirm active rickets cases using the ten point Thacher scoring system.

After diagnosis, the children were referred to the study team. The criteria for recruitment of cases were age between 1.0 and 10.9 years and this being their first presentation of suspected rickets. Children on current medication or dietary supplements which may affect bone metabolism, with known renal or intestinal disease, or other conditions causing physical disability and impaired mobility were excluded. A local control child for each case, matched for sex, village of residence and as closely as possible to age, was recruited using the Chakaria demographic surveillance survey [15]. The parents/guardians gave written informed consent, after an explanation in their native language, and they assented to the study procedures. Study visits and data collection were conducted within two weeks of recruitment and any treatment for rickets was deferred until these had been completed. All participants had two study visits: the first at the SARPV clinic in Chakaria, and the second at their home within a few days. Each case and their respective control were seen on the same day.

### 2.2 Sample size

Sample size calculation was based on detecting a difference in urinary phosphate excretion between rachitic subjects and their controls. In absence of Bangladeshi reference values for children without rickets, Gambian reference values of urinary phosphate excretion in fasting 2-hour urine were used (mean (SD): 0.27 (0.10) mmol and 0.49 (0.16) mmol in fasting 2-hour urine for 2–6 year and 6–10 year old children respectively) [8]. A sample size of 64 cases and 64 controls was required to detect a minimum group difference of 0.50 SD at a significance of 0.05 and power of 80%.

### 2.3 Clinic visit at SARPV

Participants, accompanied by their mother or carer, arrived at SARPV early in the morning having fasted overnight. After passing urine, they commenced a timed urine collection for approximately 2 h. Each participant drank a measured volume of water (200 or 300 mL for <5 or > 5 year olds respectively) over the 2 h duration and all urine passed during this time was collected directly into acid-washed containers or urine bags (Romsons, Paediatric Urine Collection Bag, DB 1062, India). One hour into the urine collection a 5 mL venous blood sample was drawn.

Anthropometry (weight, standing and sitting heights, wrist and mid-upper arm circumferences, wrist width) was performed using electronic weighing scales (Uniscale, UNICEF), a portable stadiometer, non-stretchable measuring tape, and Vernier caliper, respectively. Because lower limb deformities make it difficult to obtain accurate height measurements, demispan (as a proxy for height) was also measured as the distance between the sternal notch and the intersection between the middle and fourth finger. After completion of the urine collection, a ^13^C-urea breath test was conducted to test for the presence of *H. pylori* infection [11,16,17]. The ^13^C-urea method has been previously described in the literature [11]. In brief, breath samples were collected in duplicate from each participant in evacuated breath collection tubes (Exetainers®, Labco limited, Bucks, UK) before and 30 min after consumption of a test meal comprising 100 ml of a 10% Polycal solution (a non-sweet flavoured carbohydrate supplement, manufacturer: Nutricia, UK) mixed with 50 mg ^13^C-urea (Cambridge isotopes, MA, USA).

### 2.4 Home visit

Participants were visited at their home by study staff and a 24 h urine sample was collected. The collection started as soon as the first morning sample was voided. During the 24 h period, all urine was collected into acid-washed containers or urine bags. Small children were supervised and assisted by the field staff or the carer after proper instruction was given. Young children who were not potty-trained or who had a possibility of bed-wetting at night were supplied with urine collection bags. The urine collected in this way was poured into the acid-washed container.

During the same 24 h, all foods consumed were weighed and recorded using electronic kitchen scales (CAMRY, model EK3052). Any leftovers and spilt foods were measured and deducted from the total amount offered. For pre-packed food bought from shops, information was obtained from labels.

Structured questionnaires were used to collect information on birth order, lifestyle, sun exposure (time spent in outdoor activity, use of sun-protection and typical clothing worn), housing, living conditions (number of family members, access to safe drinking water and to a latrine, whether the child attends school and the type of school), socioeconomic status (parents' literacy and educational status, occupation, number of earning family members, dependency ratio, assets and land ownership) and history of illness. These questionnaires were based on existing national Bangladeshi nutrition and dietary surveys [13]. Information on current health status, orthopaedic complaints and dental health were collected and presence of dental signs for endemic fluorosis were checked (mottling: white patches on the teeth).

### 2.5 Sample handling, preparation, processing, storage and shipping

The venous blood sample was drawn into pre-cooled lithium-heparin (LH, 3.8 mL) and ethylenediaminetetraacetic acid-coated (EDTA, 1.2 mL) tubes and placed on ice. Residual whole blood from the needle was used to measure haemoglobin (Sahli haemometer, India, method: visual quantification of the colour of this chromoprotein) at the point of care and to prepare blood films fixed and stained with Littmann's stain (later assessed at the Haematology Department, Addenbrooke's Hospital, Cambridge, UK, for cell size and signs of microcytic and hypochromic anaemia). Plasma was separated within 1 h of collection in a refrigerated centrifuge at 3000 rpm for 15 min. Plasma aliquots were frozen immediately, stored temporarily in the clinic at −20 °C, and then transported on dry ice and stored at −70 °C at ICDDRB in Dhaka, Bangladesh.

Urine samples collected during the clinic and home visits (2 and 24 h) were kept chilled in a cool box with ice packs before being transferred to a refrigerator. The total volumes were measured, and acidified (10 μL of concentrated hydrochloric acid per mL of urine) and non-acidified aliquots were stored at −20 °C. Breath samples were stored at room temperature.

After completion of the study all samples were shipped for analysis to MRC Human Nutrition Research (subsequently known as the MRC Elsie Widdowson Laboratory), Cambridge, UK. Frozen plasma and urine samples were shipped on dry ice and breath samples and blood films at room temperature.

### 2.6 Analysis of samples

EDTA-plasma samples were used for analysing intact PTH and cFGF23 and heparinised samples for the remaining plasma analytes. Intact PTH was measured by immunometric assay (Immulite 1000 chemiluminescence, Siemens Health Care, Diagnostics Products Ltd., Gwynedd, UK). Plasma 25OHD and bone alkaline phosphatase (BALP) were measured by chemiluminescence immunoassay (Liaison; DiaSorin, Stillwater, MN, USA) and 1,25(OH)_2_D by radioimmunoassay (IDS Ltd., Boldon, Tyne and Wear, UK). Intact FGF23 (iFGF23) was measured at the VU University Medical Center, Amsterdam, The Netherlands using the Kainos ELISA kit (Tokyo, Japan). cFGF23 was analysed using a 2nd generation C-terminal, two-site enzyme-linked immunosorbant assay (Immutopics Inc., San Clemente, CA). The level of detection for the iFGF23 assay was 5 pg/mL; a nominal value of 2.5 pg/mL was used for concentrations below this threshold.

The following methods in the Dimension Xpand Clinical Chemistry System (Siemens Healthcare Diagnostics, Camberley, UK) were used to determine plasma analytes: total alkaline phosphatase (TALP), *p*-nitrophenyl phosphate method; calcium (pCa), o-cresolphthalein complexone; phosphate (pPhos), phosphomolybdate; magnesium (pMg), methylthymol blue; zinc (pZn), chelation with reagent 2-(5-bromo-2-pyridylazo)-5-(N-propyl-N-sulfopropylamino)-phenol; creatinine (pCr), Jaffé; albumin, bromocresol purple; bilirubin, modified diazo; aspartate transaminase (AST), enzymatic transamination of l-aspartate, and ferritin, automated immunoassay. Soluble transferrin receptor (sTfR) was measured by ELISA Ramco Laboratories, Inc., Stafford, TX, USA, C-reactive protein (CRP) and α1-acid glycoprotein (AGP) were analysed by particle enhanced turbidimetric immunoassay (Dimension Siemens ProSpec).

Urinary (u) uCa, uPhos, uMg and uCr were measured in acidified samples using the same colorimetric methods as used for plasma. Zinc (Zn), iodine (I), arsenic (As), cadmium (Cd), lead (Pb), mercury (Hg), molybdenum (Mo), manganese (Mn), and iron (Fe) were measured in acidified 24 h urine aliquots by inductively coupled plasma mass spectrometry (ICP-MS, Elan DRCPlus Perkin Elmer Sciex, Shelton CT). 2 h fasted urinary data are presented as ratios to uCr; 24 h urinary data are presented both as total output per 24 h and as the ratio to creatinine. For determining the presence of *H. pylori* infection, the ^13^C concentration of respiratory CO_2_ was measured by isotope ratio mass spectrometry (IRMS, AP2003 IRMS, Analytical Precision Products, Ltd., Cambridge, UK).

All assays except for PTH were performed in duplicate. Performance was monitored using kit and in-house controls. Quality assurance for 25OHD and 1,25(OH)_2_D was performed as part of the Vitamin D External Quality Assessment Scheme (www.deqas.org). The DiaSorin Liaison chemiluminescent immunoassay for 25OHD was standardised within the Vitamin D Standardisation Program (VDSP) to internationally accepted reference methods performed at NIST and the University of Ghent (https://ods.od.nih.gov/Research/vdsp.aspx). Quality assurance for PTH assays was performed as part of the National External Quality Assessment Scheme (www.ukneqas.org.uk).

### 2.7 Dietary data analyses

A bespoke version of the DINO (Diet In Nutrients Out) in-house dietary assessment platform at MRC Human Nutrition Research [18] using composition data from Bangladeshi and Indian foods [[19], [20], [21]] was used to determine nutrient intake. To judge the level of calcium intake relative to recommendations, the average intake in each group was compared with the UK Reference Nutrition Intake (RNI) for 1–10 year olds (350-550 mg/d) and the Recommended Daily Allowance (RDA) of Indian children for 1–9 year olds (600 mg/d) [22].

### 2.8 Sub-classification of rickets and control groups

The ‘Thacher Scoring Method’ was used by one of the authors (JP) to grade radiographic images of both wrists (radius and ulna) and knees (femur and tibia). Each radiograph was given a score based on the presence and extent of widening of the growth plate, degree of lucency and metaphyseal irregular margins [14].

The Thacher score was used to classify cases into those with active rickets (children with a score ≥1.5) and those with rickets-like bone deformities (score <1.5).

### 2.9 Statistical analyses, calculations and reference thresholds

DataDesk 6.3.1 (Data Description Inc., Ithaca, NY) was used. For normally distributed continuous variables, mean (standard deviation (SD)) values, are presented. For positively skewed variables, geometric mean (geometric mean-1SD, geometric mean+1SD) values are given. These were obtained by back-transforming the mean, mean-1SD and mean+1SD of the data transformed to natural logarithms. Discrete variables, and those analytical variables with a substantial number of values below the limit of detection (iFGF23) are presented as median (25th percentile, 75th percentile). Statistical analyses compared cases with their linked controls using paired Student's *t*-test, paired sign test and McNemar's ϰ^2^ test for paired data, as appropriate. A *p* value of ≤0.05 was considered statistically significant. As none of the plasma biochemical analytes were significantly different by sex, sex was not adjusted for in any of the statistical analysis.

Creatinine clearance (Ccr), as a marker of glomerular filtration rate (GFR), was calculated by Ccr = (uCr × volume of urine) / plasma Cr [23].

Renal tubular maximal reabsorption of Phos and Ca per unit of GFR (TmP/GFR and TmCa/GFR) were calculated as follows:

Tubular reabsorption of phosphate (TRP) = 1 − ((uPhos/pPhos) × (pCr/uCr)) [24],

If TRP ≤ 0.86 then TmP/GFR = TRP × pPhos mmol/L, or

if TRP > 0.86 then TmP/GFR = (0.3 × TRP/(1 − (0.8 × TRP)) × pPhos mmol/L

TmCa/GFR = ((0.56 pCa) − (uCa/Cr) × pCr)) / [1–0.08 log_e_(0.56 pCa − (uCa/Cr) × pCr))] mmol/L of glomerular filtrate (GF) [25].

The WHO AnthroPlus software was used to calculate age and sex-matched standard-deviation scores (*Z*-scores) for weight and height (https://www.who.int/growthref/tools/en/).

### 2.10 Biochemical reference thresholds

TALP, PTH, 1,25(OH)_2_D, pCa and pPhos were considered high or low when values were greater or less than the ranges provided by the assay manufacturers for children. The values were TALP ≥300 U/L (high) and ≥ 960 U/L (severe); PTH >58 ng/L (high), pCa <2.25 mmol/L (low), pPhos ≤1.45 mmol/L for children <5y and ≤1.20 mmol/L for older children (low). For 1,25(OH)_2_D >110 pmol/L, the upper limit of normal in adults, was used (high).^.^

25OHD concentrations <25 nmol/L were considered low as the value is associated with an increased risk of vitamin D deficiency and rickets [26]. Concentrations <12.5 nmol/L were regarded as hypovitaminosis D as seen in vitamin D deficiency rickets [27].

For iFGF23, a concentration ≥51.9 pg/mL was considered elevated and <5 pg/mL was considered suppressed. A cFGF23 concentration >125 RU/mL was considered elevated. These thresholds were based information provided by the assay manufacturers.

Anaemia was defined by haemoglobin (Hb) concentration <110 g/L in children <5 years of age and <115 g/L in children >5 and <11.99 years of age [28]. Iron deficiency was defined by ferritin concentration <12 and <15 μg/L for children of <5 and >5 years of age respectively [28] and/or sTfR concentration ≥8.5 mg/L [29]. Iron deficiency anaemia was defined by a low Hb concentration and the presence of microcytic and hypochromic red blood cells on blood slides.

Iodine status of each group was judged using the median (25th, 75th percentile) urinary iodine concentration (UIC) in 24 h samples against WHO criteria: Adequate nutrition 100–199 μg/L; mild deficiency 50–99 μg/L, moderate deficiency 20–49 μg/L, severe deficiency <20 μg/L [30].

A ^13^C:^12^C >5.47‰ at 30 min was regarded as positive for *H. pylori* infection [31]. Inflammation was defined as a CRP >5 mg/L [28,32].

## 3 Results

### 3.1 Participant characteristics and anthropometry

64 cases and 64 controls were recruited. One case had active rickets, a maximum Thacher score of 10, severe hypovitaminosis D and a biochemical profile substantially different from all other cases (see supplementary information). A second case had deformities and radiological features of Blount disease. In order to investigate potential aetiological factors among the remaining cases, they were categorised into groups with and without radiographically active rickets. Groups A (*n* = 24, Thacher score [median (25th percentile, 75th percentile) = 6 (3,10)], and B (*n* = 38, Thacher score [median (25th percentile, 75th percentile) = 0 (0,0)]. 75% of Group A had *genu varum* (bow-legs) and 25% had *genu valgum* (knock-knees). In Group B the bone deformities were 45% *genu varum* and 55% *genu valgum*. Other rickets signs, i.e. swollen wrists, rib beading and bossed forehead, were also present (Group A 96%, 91% and 86%; Group B 76%, 87% and 57% respectively).

The age and anthropometric variables in Groups A and B and their respective controls (AC, BC) are given in Table 1. The age of the participants ranged from 1.0 to 10.6 years. All the children in Group A were <6 years old and were, on average, 5 months younger than their controls. There were equal numbers (12) of boys and girls in Group A. Group B children were younger than their controls and there were more boys than girls (28 and 10 respectively). Group A children were on average lighter, shorter and had lower MUAC than their controls; they also had shorter demispan and sitting height. Similar differences in anthropometry were seen between Group B and their controls. Children in Groups A and B started walking later than their controls (Group A: 20 v 13 months, *p* ≤ 0.0001, Group B: 18 v 14 months, *p* = 0.04, Supplementary Table 1).

**Table 1:** Age and anthropometry of the participants by group.

Variable	Group A			Group B		
	Cases *n* = 24	Controls *n* = 24	*p*	Cases *n* = 38	Controls *n* = 38	*p*
Age (y)	2.7 (2.1, 3.4)	3.2 (2.5, 4.3)	0.04	3.0 (1.9, 4.3)	3.2 (2.4, 5.1)	0.0006
Weight (kg)	9.7 (1.7)	11.6 (2.6)	0.004	11.4 (2.7)	12.9 (3.9)	0.05
Height (cm)	78.8 (7.4)	90.4 (9.6)	≤0.0001	86.7 (10.1)	94.4 (14.3)	0.008
Weight for age *Z*-score^a^	−2.99 (1.10)	−2.11 (1.13)	0.006	−2.28 (1.01)	−1.88 (1.20)	0.1
Height for age Z-score^a^	−4.18 (1.50)	−1.90 (1.21)	≤0.0001	−2.67 (1.77)	−1.71 (1.44)	0.003
Sitting height (cm)	46.8 (3.6)	53.5 (7.1)	0.0003	50.2 (5.1)	54.5 (6.8)	0.0002
Demispan (cm)	36.9 (4.0)	42.4 (5.8)	0.002	40.3 (4.8)	43.5 (6.4)	0.05
MUAC (mm)	140 (7)	147 (11)	0.05	145 (11)	148.0 (11)	0.7

Data are described by mean (standard deviation) except age median (25th centile, 75th centile).

Group A: active rickets, Group B: rickets-like bone deformities, MUAC: mid-upper arm circumference. *p*-value indicates the significance of difference between cases and their linked controls in Groups A and B.

a Calculated from WHO growth standards.

98% of the participants were Muslim and the rest were Hindu. Consanguineous marriage (marriage between two closely connected blood relatives or first/second cousins) was found in 3% of the families (data not shown). Children in Group A were mostly firstborns (63%) with a considerable proportion of them being the only child in the family at the time of the study (33%) (Supplementary Table 1). The controls for Group A were less likely to be firstborn (21%) and only a small proportion were the only child in the family (8%). However, no group differences were seen in family size, dependency ratio, parental income, parental education or years of residence in the area (Supplementary Table 1). No differences in sun exposure were found between cases and controls in estimated time spent outdoors or use of sunscreen (Supplementary Table 1) or in customary dress (data not shown). Clothing was similar for boys and girls, mostly short-sleeved shirts, shorts/skirts worn above the knee and no head covering or hijab. No differences were found in history of diarrhoea, pneumonia, malaria, anaemia, jaundice or dental abnormalities between cases and controls (data not shown). No dental signs of endemic fluorosis were observed in any study participant.

### 3.2 Dietary intake

Mean dietary calcium intakes were low in all groups compared to the UK and Indian reference values (Table 2) and were 50% lower in Group A children than their controls (*p* = 0.005). Dietary phosphorus intake, the Ca:P ratio and energy intake were also lower in Group A compared to controls. Dietary calcium and phosphorus intakes expressed relative to total energy intake showed a similar pattern.

**Table 2:** Dietary calcium, phosphorus and energy intakes of the participants by group.

Variable	Group A			Group B		
	Cases *n* = 24	Controls *n* = 24	*p*	Cases *n* = 38	Controls *n* = 38	*p*
Calcium mg/d	156 (80)	323 (249)	0.005	239 (166)	254 (222)	0.7
Phosphorus mg/d	322 (113)	424 (155)	0.009	380 (158)	403 (168)	0.3
Ca:P (mg/mg)	0.49 (0.23)	0.74 (0.53)	0.03	0.64 (0.37)	0.61 (0.34)	0.7
Energy Kcal/day	847 (197)	961 (314)	0.05	905 (388)	992 (358)	0.2
Calcium/energy^a^	185 (86)	345 (266)	0.007	288 (203)	249 (160)	0.4
Phosphorus/energy^a^	378 (74)	446 (103)	0.02	427 (88)	405 (77)	0.2

Normally distributed data, the results are mean (SD). Group A: active rickets, Group B: rickets-like bone deformities. Paired *t*-tests were used to calculate *p*-values for differences between cases and controls in Groups A and B.

a Calcium/energy and phosphorus/energy = calculated values for calcium and phosphorus intakes in mg/1000 kcal of energy intake.

### 3.3 Biochemistry

TALP, BALP, 1,25(OH)_2_D and PTH concentrations were higher in Groups A and B compared to their controls (Table 3); Group A were affected to a greater extent than Group B, *p* ≤ 0.0001. Many children had elevated TALP (≥300 U/L) and PTH (>58 ng/L), especially among the cases (Table 4), and all participants had a 1,25(OH)_2_D concentration >110 pmol/L. Plasma 25OHD, pCa and pPhos concentrations were lower in Group A compared to their controls but not to Group B. The mean 25OHD concentration in Group A was half that of their controls (*p* ≤ 0.0001); 62.5% of Group A had a 25OHD concentration <25 nmol/L, of which only 4% (*n* = 1) had a concentration <12.5 nmol/L, compared to 8% of their controls with a concentration <25 nmol/L and none <12.5 nmol/L (Table 4). There was no significant difference in mean 25OHD concentration between Group B cases and controls, with 13% and 5% respectively <25 nmol/L and none <12.5 nmol/L. Group A had lower pCa and pPhos compared to their controls (*p* = 0.004 and ≤0.0001 respectively) with 83% below the normal range for their age for phosphate compared with 22% of controls (*p* ≤ 0.0001, Table 4). Concentrations of pCa were low in all groups with a high proportion of participants below the assay-specific normal range for children (Table 4). Adjustment for albumin did not alter this picture.

**Table 3:** Blood biochemistry of the participants by group.

Variable	Group A			Group B		
	Cases *n* = 24	Controls *n* = 24	*p*	Cases *n* = 38	Controls *n* = 38	*p*
TALP (U/L)*	595 (372, 953)	234 (172, 318)	≤0.0001	265 (186, 379)	214 (147, 313)	0.02
BALP (μg/L)*	265 (156, 450)	81 (52, 125)	≤0.0001	93 (58,151)	75 (54, 106)	0.03
PTH (ng/L)*	158.7 (53.0, 475.0)	44.0 (24.2, 80.0)	≤0.0001	50.9 (24.3, 106.8)	27.7 (10.2, 74.7)	0.002
1,25(OH)_2_D (pmol/L)*	479 (356, 646)	288 (214, 389)	≤0.0001	363 (249, 529)	311 (229, 423)	0.05
25OHD (nmol/L)	23.7 (7.87)	44.9 (12.0)	≤0.0001	42.8 (18.9)	48.6 (16.5)	0.2
pCa (mmol/L)*	1.90 (1.59, 2.27)	2.07 (1.84, 2.34)	0.004	2.13 (1.89, 2.41)	2.18 (1.97, 2.41)	0.9
pPhos (mmol/L)*	1.16 (0.87, 1.55)	1.62 (1.37, 1.90)	≤0.0001	1.64 (1.35, 2.00)	1.63 (1.40, 1.89)	0.7
pMg(mmol/L)*	0.85 (0.74, 0.98)	0.85 (0.75, 0.97)	0.9	0.91 (0.78, 1.06)	0.89 (0.78, 1.01)	0.3
pZn (μmol/L)*	12.4 (10.5, 130.6)	11.6 (9.94, 115.0)	0.2	13.1 (10.8, 142.0)	12.2 (10.1, 123.0)	0.7
pCr (μmol/L)*	14.5 (10.4, 20.2)	19.8 (13.6, 28.9)	0.006	19.6 (13.7, 27.9)	20.8 (15.5, 27.7)	0.4
Albumin (g/L)	40.5 (6.61)	39.4 (6.38)	0.4	40.9 (5.79)	40.1 (4.76)	0.4
iFGF23 (pg/mL)§	2.5 (2.5, 2.5)	22.2 (16.5, 26.9)	≤0.0001	18.9 (5.5, 29.0)	24.5 (14.8, 30.7)	0.4
cFGF23 (RU/mL)*	34.2 (15.7, 74.5)	41.8 (22.8, 76.6)	0.3	44.4 (22.5, 87.3)	45.2 (24.9, 82.2)	0.9
Hb (g/L)	102 (8.4)	102 (8.0)	0.9	103 (6.8)	103 (9.0)	0.8
Ferritin (μg/L)*	33.0 (19.2, 56.6)	27.7 (16.8, 45.7)	0.3	32.8(15.5, 69.4)	31.3(16.2, 60.3)	0.7
sTfR (mg/L)*	5.46 (4.48, 6.66)	5.17 (4.03, 6.63)	0.4	5.30 (3.97, 7.07)	4.69 (3.74, 5.88)	0.03
CRP (mg/L)*	2.22 (0.87, 5.67)	2.07 (1.16, 3.68)	0.5	2.20 (1.25, 3.87)	2.02 (1.24, 3.29)	0.6
AGP (g/L)*	0.67 (0.45, 1.00)	0.76 (0.53, 1.09)	0.2	0.82 (0.57,1.18)	0.75 (0.49, 1.14)	0.1
Bilirubin (μmol/L)*	2.86 (2.21, 3.69)	3.27 (2.37, 4.52)	0.1	3.28 (2.36, 4.55)	3.49 (2.34, 5.20)	0.5
AST (U/L)*	38.3 (29.3, 50.0)	38.1 (27.9, 51.9)	0.9	38.4 (30.5, 48.3)	33.9 (22.1, 51.9)	0.07

For normally distributed data, the results are mean (standard deviation); for positively skewed data (denoted by *) the results are geometric mean (geometric mean-1SD, geometric mean + 1SD); for iFGF23 (denoted by §) with substantial proportion below the level of detection of the assay (given a nominal value of 2.5 pg/mL) the results are median (25th centile, 75th centile). Group A: active rickets, Group B: rickets-like bone deformities. TALP and BALP: Total and bone specific alkaline phosphatase respectively, PTH: Parathyroid hormone, 1,25(OH)_2_D: 1,25-dihydroxyvitamin D, 25OHD: 25-hydroxyvitamin D, pCa: Calcium; pPhos: Phosphate, pMg: Magnesium, pZn: Zinc, pCr: Creatinine, cFGF23 and iFGF23: c-terminal and intact- fibroblast growth factor respectively, Hb: Haemoglobin, sTfR: Serum transferrin receptor, CRP: C-reactive protein, AGP: α1-acid glycoprotein and AST: aspartate transaminase. Paired *t*-tests were used to calculate *p*-values for differences between cases and controls in Groups A and B with variables transformed to natural logarithms, except for iFGF23 for which the non-parametric paired sign test was used.

**Table 4:** Percentage of participants with blood values above or below accepted norms or the presence of *H. pylori* infection by group.

Variable	Group A			Group B		
Threshold used for status	Cases *n* = 24	Controls *n* = 24	*p*	Cases *n* = 38	Controls *n* = 38	*p*
TALP (%) High: ≥300 U/mL	92	17	≤0.0001	42	16	0.01
TALP (%) Severe: ≥960 U/mL	8	0	0.2	0	0	–
PTH (%) High: >58 ng/L	88	25	≤0.0001	42	26	0.2
25OHD (%) Low: <25 nmol/L	63	8	≤0.0001	13	5	0.2
25OHD (%) Severe <12.5 nmol/L	4	0	0.3	0	0	–
pCa (%) Low: ≤2.25 mmol/L	91	88	0.9	72	75	0.6
pPhos (%) Low: ≤1.45 or ≤1.20 mmol/L^a^	83	22	≤0.0001	16	14	0.8
iFGF23 (%) Low: <5 pg/mL	88	4	≤0.0001	21	0	0.006
iFGF23 (%) High: ≥51.9 pg/mL	0	0	–	0	0	–
cFGF23 (%) High: >125 RU/mL	4	8	0.6	8	3	0.3
Hb (%) Low: <110 or <115 g/L^a^	67	71	0.8	76	74	0.8
Ferritin (%) Low: ≤10 or ≤15 μg/L^a^	4	8	0.6	5	8	0.6
CRP (%) High: ≥5 mg/L	15	7	0.5	11	5	0.4
Iron deficiency anaemia (%)	8	17	0.4	18	21	0.8
*H. pylori* infected (%)	29	58	0.04	49	51	0.5

Group A: active rickets, Group B: rickets-like bone deformities. McNemar ϰ^2^ test *p*-values are presented. ^a^Threshold values for <5 and >5 year old children. TALP: Total alkaline phosphatase, PTH: Parathyroid hormone, 25OHD: 25-hydroxyvitamin D, pCa: Calcium; pPhos: Phosphate, iFGF23 and cFGF23: intact- and c-terminal fibroblast growth factor, Hb: Haemoglobin, sTfR: Serum transferrin receptor, CRP: C-reactive protein, Iron Deficiency Anaemia: Low haemoglobin plus microcytic and hypochromic red blood cells.

iFGF23 was lower in Groups A and B compared to their controls: 88% of Group A and 21% of Group B compared to 4% (*n* = 1) of Group A controls and none of Group B controls had concentrations below the limit of detection (Table 4). The children in Groups A and B with undetectable iFGF23 had lower pCa, pPhos, TmP/GFR, 25OHD and higher TALP than the other cases in their group (data not shown). No group differences were seen in cFGF23 concentration; elevated cFGF23 was seen in 4% of Group A compared with 8% of their controls and in 8% of Group B compared with 3% of their controls. No child had an elevated iFGF23 concentration.

Hb was low in all groups and there were no group differences (Table 3). More than 60% of the children were anaemic but only 4–8% of children had low ferritin and a similar proportion had elevated sTfR concentration (Table 4), although no child had both low ferritin and raised sTfR concentrations. These proportions did not differ by group. Blood film analysis also indicated that only a small percentage of children had iron deficiency anaemia as defined by low haemoglobin and microcytic and hypochromic red blood cells (Table 3, Table 4). None of the films indicated the occurrence of megaloblastic anaemia (usually caused by folate deficiency) and it was not possible to diagnose other anaemias from the blood films. Ferritin was a significant negative predictor of both iFGF23 and cFGF23 concentration, independent of group (*p* ≤ 0.0001), relationships that remained after adjustment for the inflammatory markers CRP and AGP.

There were no differences between the groups to suggest involvement in the aetiology of rickets in CRP or AGP, poor liver function (bilirubin, AST) or *H. pylori* infection, although elevated values were seen in a number of cases and control children (Table 4).

Table 5 presents the 2 h and 24 h urinary results for the bone-forming minerals. For calcium, the 2 h fasted samples revealed a lower excretion threshold in Group A compared with controls (TmCa/GFR 1.96 v 2.13 mmol/L, *p* = 0.02) indicating the potential for greater calcium loss, although their daily urinary calcium output was lower by 50% (*p* ≤ 0.0001) reflecting their lower calcium intake. Correcting for their lower 24 h urinary creatinine output (Table 5), a likely consequence of their lower weight, reduced the difference in daily calcium output and it was no longer significant. A similar pattern was observed for zinc, measured only in 24 h samples (Supplementary Table 2). There was evidence of greater phosphate excretion in Group A compared to their controls in the 2 h fasted samples (uPhos:Cr ratio 5.52 v 3.73 mol/mol, *p* = 0.009; TmP/GFR 1.20 v 1.92 mmol/L, *p* ≤ 0.0001) and in the 24 h collections after creatinine correction (uPhos:Cr ratio 5.00 v 3.36 mol/mol, *p* ≤ 0.0001). The pattern of differences between cases and controls for urinary calcium, zinc and phosphate was similar in Group B but attenuated in size and significance. There were no significant differences in 2 h or 24 h urinary magnesium between cases and controls in either group.

**Table 5:** Urinary excretion of bone-forming minerals of the participants by group.

Variable	Group A			Group B		
	Cases *n* = 24	Controls *n* = 24	*p*	Cases *n* = 38	Controls *n* = 38	*p*
**2 hour fasted urine**						
*u*Ca:Cr mol/mol*	0.67 (0.21, 2.15)	0.62 (0.24, 1.64)	0.6	0.61 (0.18, 2.08)	0.71 (0.27, 1.83)	0.6
uPhos:Cr mol/mol*	5.52 (3.50, 8.69)	3.73 (2.31, 6.01)	0.009	4.79 (2.28, 10.1)	3.60 (1.92, 6.77)	0.04
uMg:Cr (mol/mol)*	0.83 (0.46, 1.50)	0.47 (0.20, 1.13)	0.1	0.68 (0.36, 1.29)	0.46 (0.19, 1.08)	0.3
TmCa/GFR mmol/L	1.96 (0.46)	2.13 (0.45)	0.02	1.93 (0.45)	1.87 (0.42)	0.08
TmP/GFR mmol/L	1.20 (0.36)	1.92 (0.39)	≤0.0001	1.88 (0.48)	1.96 (0.46)	0.3
*u*Cr mmol/2 h*	0.036 (0.017, 0.077)	0.055 (0.025, 0.119)	0.009	0.059 (0.018, 0.194)	0.075 (0.302,0.187)	0.4
Ccr mL/min*	21.3 (8.72, 52.2)	23.0 (9.60, 55.1)	0.6	25.2 (7.48, 84.9)	29.9(11.8, 75.7)	0.6

**24hour urine output**						
uCa mmol/24 h	0.15 (0.06, 0.36)	0.31 (0.14, 0.66)	≤0.0001	0.29 (0.13, 0.64)	0.41 (0.18, 0.94)	0.02
uPhos mmol/24 h	2.56 (1.29, 5.08)	2.93 (1.41, 6.11)	0.5	3.17 (1.83, 5.49)	3.41 (1.88, 6.19)	0.5
uMg mmol/24 h	0.58 (0.29, 1.15)	0.81 (0.41, 1.59)	0.08	0.78 (0.41, 1.49)	0.86 (0.46, 1.61)	0.3
uCr mmol/24 h*	0.51 (0.27, 0.96)	0.87 (0.48, 1.58)	0.001	0.75 (0.46, 1.22)	0.96 (0.53, 1.75)	0.008
uCa:Cr (mol/mol)*	0.29 (0.13, 0.69)	0.35 (0.17, 0.75)	0.2	0.39(0.19, 0.81)	0.42 (0.20, 0.89)	0.6
uPhos:Cr (mol/mol)*	5.00 (3.73, 6.72)	3.36 (2.35, 4.81)	≤0.0001	4.22 (3.13, 5.69)	3.54 (2.52, 4.97)	0.02
uMg/Cr (mol/mol)	1.13 (0.69, 1.84)	0.93 (0.60, 1.83)	0.1	1.04 (0.66, 1.64)	0.89 (0.53, 1.50)	0.1
Ccr mL/min*	24.0 (11.4, 50.4)	30.5 (15.3, 60.8)	0.2	26.6 (16.2, 43.6)	32.3 (18.7, 55.6)	0.05
uVolume (mL/24 h)	436 (252)	496 (212)	0.2	497 (264)	595 (342)	0.06

For normally distributed data, the results are mean (standard deviation); for positively skewed data (denoted by *) the results are geometric mean (geometric mean-1SD, geometric mean + 1SD). Group A: active rickets, Group B: rickets-like bone deformities, u: urinary, Ca: Calcium, Phos: Phosphate, Mg: Magnesium, Cr: Creatinine, TmCa/GFR and TmP/GFR: Tubular maximum calcium or phosphate reabsorption per unit of glomerular filtrate rate respectively, Ccr: Creatinine clearance. Paired t-tests were used to calculate *p*-values for differences between cases and controls in Groups A and B with variables transformed to natural logarithms.

The 24 h urine data indicated that iodine status was poor in all groups with the urinary iodine concentration (UIC) indicating moderate to severe iodine deficiency (median [25th–75th percentile]: Group A cases = 22 [[12], [13], [14], [15], [16], [17], [18], [19], [20], [21], [22], [23], [24], [25], [26], [27], [28], [29], [30], [31], [32], [33], [34], [35], [36], [37], [38], [39], [40]] μg/L, controls 22 [[19], [20], [21], [22], [23], [24], [25], [26], [27], [28], [29], [30], [31], [32], [33], [34], [35], [36], [37], [38], [39], [40], [41], [42], [43], [44]] μg/L; Group B cases 37 [13–81] μg/L, controls 29 [[15], [16], [17], [18], [19], [20], [21], [22], [23], [24], [25], [26], [27], [28], [29], [30], [31], [32], [33], [34], [35], [36], [37]] μg/L). The daily iodine output was significantly lower in Group A rickets children but not when expressed relative to creatinine (Supplementary Table 2). Total daily urinary metals output did not differ significantly between cases and controls in either group (Supplementary Table 3). However, after adjusting for creatinine, metals excretion was greater in Group A compared to controls, significantly so for all metals except arsenic.

## 4 Discussion

Bangladesh is a country in South East Asia (latitude 21°N) with a hot and tropical climate. Despite the presence of sunshine throughout the year, rickets is of public health concern with an estimated prevalence of 2.1% among children 1–15 years old in Chakaria, Southern Bangladesh [13]. This study has undertaken a thorough investigation of the aetiology of rickets in Chakarian children by considering nutritional status, lifestyle, dietary intake, biochemical profile, exposure to metals and *H. pylori* infection compared to age-matched controls from the same district.

This has shown that children with active rickets (Group A) weighed less, were smaller and had substantially lower calcium intake, lower 25OHD concentration and higher urinary metals excretion relative to creatinine compared to their controls. Group A had the characteristic biochemical profile of children with active calcium-deficiency rickets of elevated TALP, PTH and 1,25(OH)_2_D concentrations, lower plasma calcium and phosphate concentrations and increased urinary phosphate loss. In general, children with rickets-like bone deformities but no radiographic evidence of active rickets (Group B) showed a similar pattern of differences to their controls but were affected to a lesser extent. One additional child with active rickets, who has been described separately, had a biochemical profile that indicated frank vitamin D deficiency, rather than dietary calcium deficiency (see Supplementary Information).

Dietary calcium intake was low in all groups, with means below 350 mg/day. This was most prevalent in Group A who consumed, on average, only 156 mg/day. Group A also had low energy intake, but calcium intake remained lower than controls after energy adjustment. The low calcium intake was reflected in low 24 h urine calcium excretion which was half that of controls. However, relative to creatinine output, which was also lower in these children, this difference was reduced and was not significant. Daily creatinine output is related to lean body mass and, although often used to correct for inaccuracies in total volume, it is also a partial correction for body weight [33]. Therefore, the urinary data suggest that the daily calcium excretion was on par with that of controls once body weight was taken into consideration. The 2 h fasted uCa to uCr ratios were quite high in all the children (>0.6 mmol/mmol; the upper limit of normal) [34]. This again relates to the very low creatinine outputs related to low lean body mass of these smaller and shorter children. Dietary phosphorus intake was also lower in Group A compared with controls, but their lower TmP/GFR resulted in greater urinary phosphate excretion relative to creatinine. The urinary data identified poor status of two other nutrients, zinc and iodine. Zinc deficiency is known to be prevalent in Bangladesh, as is poor iodine status despite the mandatory iodization of salt in the country [35,36]. This points to the diet of these children being particularly poor in many respects compared to their peers, and more research is needed to consider the contribution of other nutrient deficiencies to rickets in this population.

Children in Group A also had lower vitamin D status than their controls and more than half had a 25OHD concentration <25 nmol/L, although only one had a value <12.5 nmol/L. Intriguingly, factors often associated with vitamin D status, i.e. lifestyle, clothing and sun exposure [37], were not significantly different between groups. Many in Group A were firstborns and >2 years old, which is older than the age at which patients with frank vitamin D-deficiency rickets often present [38,39]. Evidence from the literature suggests that a low calcium intake can reduce 25OHD concentration through increasing its conversion to active and inactive metabolites [11]. It is possible, therefore, that rather than indicating an inadequate vitamin D supply, the lower 25OHD concentrations of Group A may be a consequence of their very low calcium intakes.

The low dietary calcium intake is likely to have resulted in the observed elevated plasma PTH and 1,25(OH)_2_D, and decreased phosphate reabsorption in the renal tubules. Such metabolic changes would normally promote release of bone mineral and increase intestinal absorption of calcium and phosphorus to regularise blood levels, but the very low calcium intakes of Group A children may have prevented this, resulting in increased urinary phosphorus excretion and rickets. Interestingly, 88% of Group A and 21% of Group B had undetectable levels of iFGF23 compared to none of the controls. The child diagnosed with hypovitaminosis D also had undetectable iFGF23.

FGF23 is a phosphate-regulating hormone, expressed in osteocytes [40]. FGF23 expression is increased in response to high plasma phosphate and 1,25(OH)_2_D concentrations [41]. FGF23 targets renal tubular cells where it causes internalisation and degradation of sodium-phosphate transporters, thereby increasing urinary phosphate loss in an attempt to reduce elevated circulating phosphate concentrations [41]. It is possible that the low plasma phosphate concentration of children in Group A was involved in a negative feedback loop with FGF23, resulting in suppression of FGF23 gene expression in order to preserve renal phosphate reabsorption. It is also possible that the low iFGF23 in many of the rickets children may have been due to their low plasma calcium, because calcium is known to be a modulator of circulating FGF23 [42]. There is evidence from rodent studies that circulating FGF23 is reduced in hypocalcaemic animals fed on a low calcium diet. This could be a protection mechanism against suppression of 1,25(OH)_2_D production by FGF23, thereby preventing the consequent reduction in intestinal absorption and skeletal release of calcium. In addition, FGF23 has been shown to be increased by a high calcium diet in parathyroidectomised and vitamin D receptor knockout animals [42,43].

Although iFGF23 concentrations were low in the rickets patients, cFGF23 concentrations were in the normal range. The intact FGF23 hormone (measured in plasma using the iFGF23 assay) undergoes cleavage into its n- and c-terminal fragments (the cFGF23 assay measures both intact hormone and the cleaved C-terminal fragment in the plasma). Following release into the circulation both iFGF23 and cFGF23 undergo further degradation. The mechanisms that control cleavage and the ratio of cFGF23 to iFGF23 in the circulation are not completely understood and may differ depending on age and disease state [[44], [45], [46], [47], [48]]. Differential stability of these proteins in stored samples may also be a possible reason for the low iFGF23-to-cFGF23 ratio in the Bangladesh samples, but the fact that undetectable iFGF23 was only seen in samples from rickets patients and that these children had significantly lower pPhos, pCa, Tmp/GFR, 25OHD, and higher TALP than the other cases in their group, suggests that this was a genuine finding and one related to the metabolic disturbances underlying rickets.

Therefore, the hypothesis that the chronically low dietary calcium intakes of children in Chakaria predisposes to urinary phosphate wasting and rickets was upheld. However, despite the resulting high 1,25(OH)_2_D concentrations, there was no evidence that the phosphaturia was driven by elevations in FGF23, unlike the situation in The Gambia.

Group A had higher urinary excretion of metals relative to creatinine compared with controls. This may reflect higher environmental exposure to these metals or may be a consequence of the greater bone turnover and loss in these children, as many of these metals are known to be stored within the skeleton [49]. These metals have known inhibitory effects on calcium absorption, and deleterious effects on the kidney and skeleton [50,51] and may therefore contribute to the development of rickets in these children. Previous studies in Chakaria in 1990s did not find any high exposure to aluminium, cadmium, lead, arsenic through food and water or through use of aluminium cooking pots [3,52,53]. In the years since this research, there have been increases in the potential for environmental pollution from many sources, such as from chemical fertilisers, tubewells and irrigation systems, industrial food packaging and automobile transportation [[53], [54], [55]]. Therefore, future studies of rickets aetiology in this region may consider measuring aluminium, cadmium, lead, arsenic in food and water in this region. In our study, we did not find any association between parents' occupation and/or participant's area of residence with either rickets or high urinary excretion of any specific environmental contaminant or metal.

Another possible link between rickets and impaired calcium absorption was through *H. pylori* infection [56]. However, although infection was detected in a number of participants, there was no evidence of a link with rickets. Another potential contributory factor is endemic fluorosis [57] but this was unlikely because no dental signs of fluorosis were observed, and, although not measured in this study, water fluoride is known to be generally low in Bangladesh [58].

The biochemical profile of the Bangladeshi children with rickets differed in a number of respects from that reported from The Gambia where calcium deficiency was also suspected [7,27]. Firstly, the vitamin D status was lower both in the cases and control children compared with The Gambia. Although these 25OHD concentrations were similar to many seen in healthy children world-wide, it is possible that they represent marginal status, predisposing to rickets, when calcium intakes are low, diet quality is poor and there is exposure to environmental metals. Secondly, and most notably, cFGF23 concentrations were not elevated compared to controls unlike the Gambian children with rickets-like bone deformities [7,8]. This may be related to the lower prevalence of iron deficiency, as defined by ferritin and serum transferrin receptor concentrations (4–8% rather than 24% in Gambian children [59]). Iron deficiency has been identified as a driver of FGF23 gene expression, possibly through hypoxia-inducible factors (HIFα), and poor iron status has been linked to reduced degradation capacity of cFGF23 fragments, both of which would lead to the high circulating concentrations of cFGF23 seen in The Gambia [8,47,59].

The major strengths of this case control study are 1) the use of defined criteria to categorise patients into active and non-active rickets, thereby covering the spectrum of the disease, 2) the comparison with control children from the local community and 3) investigation of a comprehensive range of potential dietary, environmental, biochemical, socioeconomic and cultural factors using appropriate methodologies. However, the study is limited because the study groups were relatively small and not fully matched for age, and it is not possible to draw conclusions about cause and effect from this observational study.

In summary, this study suggests a chronically low dietary calcium intake is the main cause of rickets in Chakaria district of Bangladesh, possibly exacerbated by marginal vitamin D status, poor diet quality and contaminated environments. The study discounted the likely influence of iron deficiency, *H. pylori* and other infections on rickets aetiology in this population. The children with rickets-like bone deformities but low Thacher scores had metabolic disturbances similar to those with active rickets, suggesting they were in the healing/healed phase of the disease and that they shared the same aetiological background. One child of the 63 patients in the study with rickets, however, exhibited the classical profile of primary vitamin D deficiency. Thus, both calcium deficiency and vitamin D deficiency need to be considered in diagnosing and treating rickets in this population. These findings increase the evidence base for the aetiology of rickets in Chakaria and for development of effective prevention and treatment strategies.

## Funding sources

This research was jointly funded by the UK 10.13039/501100000265Medical Research Council (MRC) and the 10.13039/501100000278Department for International Development (DFID) under the MRC/DFID Concordat agreement (MRC Programme number U105960371). Sonia Ahmed was in receipt of a University of Cambridge Islamic Development Bank PhD Studentship.

## Prior publication

Summary data were presented as abstracts and poster at the 8th International Symposium on Nutritional Aspects of Osteoporosis (ISNAO), Lausanne Switzerland 2012 and the 20th International Congress of Nutrition (IUNS), Granada Spain 2013.

## CRediT authorship contribution statement

**Sonia Ahmed:** Conceptualization, Investigation, Methodology, Project administration, Data curation, Formal analysis, Funding acquisition, Writing - original draft, Writing - review & editing. **Gail R. Goldberg:** Supervision, Conceptualization, Project administration, Writing - original draft, Writing - review & editing. **Rubhana Raqib:** Conceptualization, Resources, Writing - review & editing. **Swapan Kumar Roy:** Conceptualization, Writing - review & editing. **Shahidul Haque:** Conceptualization, Resources, Writing - review & editing. **Vickie S. Braithwaite:** Conceptualization, Formal analysis, Writing - review & editing. **John M. Pettifor:** Conceptualization, Writing - review & editing. **Ann Prentice:** Conceptualization, Supervision, Formal analysis, Writing - review & editing, Funding acquisition.

## Declaration of competing interest

None.

## Appendix A Supplementary information and data

Supplementary material Image 1
