# Supplementary material for: Aetiology of nutritional rickets in rural Bangladeshi children
Source: Bone. 2020 Jul;136:115357. doi: 10.1016/j.bone.2020.115357 (PMC7262584; doi:10.1016/j.bone.2020.115357)
Supplement: Supplementary file 1 — Supplementary material [file mmc1.docx]

**Supplementary Information**

**Information on the child considered separately due to hypovitaminosis D:**

This child had active rickets and was a four-year-old girl. She had a very low 25OHD concentration which was below the detectable range (<4 nmol/L), very low 1,25(OH)_2_D (61.5 pmol/L), pCa (1.45 mmol/L) and pPhos (0.37 mmol/L), very high PTH (779 ng/L), and the highest TALP among all the study children (1541 U/L). The child had a normal cFGF23 (30.4 RU/ml) and undetectable iFGF23. This child also had a low fasting uCr (0.04 mmol/2h), very high uPhos:Cr (12.8 mol/mol), uCa/Cr (10.8 mol/mol), high uMg:Cr (2.5 mol/mol) and a very low TmP:GFR (0.16 mmol/GF). In 24 hour excretion, she had a lower total uCr output (0.18 mmol/24h), a very low Ccr (7.5 ml/min), and high uPhos:Cr (5.16 mol/mol), uCa/Cr (1.47 mol/mol), and uMg:Cr (3.18 mol/mol) compared to other children (Table 4).

**Supplementary Table 1:** Characteristics of the study participants by group

| Variable | Group A | |  | | |  |  | | Group B | | | | |  | | |  |  |  |  |  |  |
| --- | --- | --- | --- | --- | --- | --- | --- | --- | --- | --- | --- | --- | --- | --- | --- | --- | --- | --- | --- | --- | --- | --- |
|  | | Cases n=24 | | Controls n=24 | *p* | | |  | Cases n=38 | | Controls n=38 | | | | | *p* | | |  |  |  |  |
| Age at first walk (months) | | | 20 (7) | | 13 (2) | ≤0.0001 | | | | | 18 (11) | | 14 (3) | | | 0.04 | | | | | |  |
| Birth order (% firstborns) | | | 62.5% | | 21% | 0.004 | | | | | 21% | | | 8% | | 0.1 | | | | | | |
| *Family characteristics* | | |  | |  |  | | | | |  | | |  | |  | | | | | | |
| No of children in family | | | 2 [1, 3] | | 3.5 [2, 4.5] | 0.02 | | | | | 3 [2, 4] | | 4 [3, 6] | | | 0.04 | | | | | |  |
| No. of family members | | | 6 [4, 7] | | 5 [4, 6.5] | 0.5 | | | | | 6 [4, 7] | | 6 [5, 8] | | | 0.2 | | | | | |  |
| Earning members | | | 1 [1,1] | | 1 [1,1] | 0.3 | | | | | 1 [1,2] | | 1 [1,2] | | | 0.5 | | | | | |  |
| Dependency ratio | | | 5.00 (1.86) | | 5.04 (1.52) | 0.9 | | | | | 4.68 (1.35) | | 5.11 (1.79) | | | 0.2 | | | | | |  |
| Income (BD Taka/month) | | | 4674 (1593) | | 3909 (859) | 0.06 | | | | | 4563(2579) | | 5300 (5448) | | | 0.4 | | | | | |  |
| Mother’s residence in the area (years) | | | 30.5 (12.0) | | 31.7 (10.0) | 0.7 | | | | | 33.8 (18.2) | | 37.5 (17.5) | | | 0.1 | | | | | |  |
| Father’s education (years) | | | 4.13 (2.66) | | 3.63 (4.11) | 0.6 | | | | | 3.03 (3.08) | | 3.78 (3.36) | | | 0.3 | | | | | |  |
| Mother’s education (years) | | | 4.92 (2.52) | | 3.92 (3.06) | 0.2 | | | | | 4.00 (2.97) | | 4.43 (3.59) | | | 0.6 | | | | | |  |
| Time in the sun (mins/day)* | | | 58.8 (57.1, 60.5) | | 63.9 (62.2, 65.7) | 0.6 | | | | | 68.5 (66.6, 70.3) | | 81.8 (80.0, 83.6) | | | 0.2 | | | | | |  |
| Use of sunscreen (%) | | | 8.3 | | 8.3 | 0.9 | | | | | 10.8 | | 13.9 | | | 0.7 | | | | |  |  |

For normally distributed continuous data, the results are mean (standard deviation) and for positively skewed data (denoted by *) the results are geometric mean (geometric mean-1SD, geometric mean+1SD). Categorical variables are median [25^th^ centile, 75^th^ centile] or percentages. Group A: active rickets, Group B: rickets-like bone deformities. Paired t-tests were used to calculate *p*-values for differences between cases and controls in Groups A and B with variables transformed to natural logarithms. McNemar ϰ^2^ test was used to calculate p-values for categorical variables.

Supplementary Table 2 Daily urinary excretion of zinc and iodine

| Variable | | Group A |  |  | |  | | Group B |  | |  | | |  |  |
| --- | --- | --- | --- | --- | --- | --- | --- | --- | --- | --- | --- | --- | --- | --- | --- |
|  | | Cases n=24 | Controls n=24 | *p* | |  | | Cases n=38 | Controls n=38 | | | | *p* | |  |
| uZn µg/24h | 65.7 (31.5, 137.1) | | 107.6 (58.8, 196.8) | | 0.0006 | | 96.2 (53.1, 174.3) | | | 124.7 (69.1, 225.1) | | 0.04 | | | |
| uZn:Cr µg/mmol | 127.1 (67.3, 240.2) | | 123.3 (83.9, 181.2) | | 0.8 | | 128.2 (82.6, 199) | | | 129.4 (81.5, 205) | | 0.9 | | | |
| uI µg/24h | 7.47 (3.05, 18.3) | | 13.1 (6.56, 26.4) | | 0.02 | | 14.4 (5.16, 40.4) | | | 13.2 (6.42, 27.2) | | 0.6 | | | |
| uI:Cr µg/mmol | 14.6 (5.89, 36.3) | | 15.0 (5.44, 41.7) | | 0.9 | | 19.2 (7.24, 51.0) | | | 13.7 (6.39, 29.3) | | 0.04 | | | |

The data were positively skewed and are presented as geometric mean (geometric mean-1SD, geometric mean+1SD). Group A: active rickets, Group B: rickets-like bone deformities, Zn: zinc, I: iodine, Cr: Creatinine. Paired t-tests were used to calculate *p*-values for differences between cases and controls in Groups A and B with variables transformed to natural logarithms.

Supplementary Table 3 Urinary excretion of the metals of the participants by group

| Variable | | Group A |  |  | |  | | Group B |  | |  | | | |  |  |  |
| --- | --- | --- | --- | --- | --- | --- | --- | --- | --- | --- | --- | --- | --- | --- | --- | --- | --- |
|  | | Cases n=24 | Controls n=24 | *p* | |  | | Cases n=38 | Controls n=38 | | | | | *p* | |  |  |
| **24h urinary output** |  | |  | |  | |  | | |  | | |  | | | | |
| uAs µg/24h | 11.2 (5.3, 23.8) | | 15.1 (7.7, 29.7) | | 0.08 | | 11.2 (5.4, 23.1) | | | 13.3 (6.3, 28.2) | | | 0.2 | | | | |
| uCd µg/24h | 1.77 (0.82, 3.84) | | 1.99 (0.80, 5.01) | | 0.5 | | 1.74 (0.76, 3.99) | | | 1.81 (0.75, 4.39) | | | 0.8 | | | | |
| uFe µg/24h | 13.4 (5.9, 30.4) | | 11.5 (5.0, 26.3) | | 0.4 | | 10.4 (4.37, 24.8) | | | 11.2 (4.9, 25.5) | | | 0.7 | | | | |
| uHg µg/24h | 0.17 (0.07, 0.42) | | 0.17 (0.06, 0.44) | | 0.9 | | 0.19 (0.07, 0.51) | | | 0.27 (0.09, 0.78) | | | 0.04 | | | | |
| uMn µg/24h | 2.40 (0.75, 7.67) | | 2.08 (0.66, 6.51) | | 0.3 | | 1.75 (0.7, 4.36) | | | 2.09 (0.75, 5.90) | | | 0.5 | | | | |
| uMo µg/24h | 28.7 (14.4, 57.2) | | 32.0 (12.6, 81.2) | | 0.6 | | 27.7 (12.2, 62.6) | | | 40.4 (16.8, 97.3) | | | 0.03 | | | | |
| uPb µg/24h | 1.09 (0.51, 2.36) | | 0.91 (0.54, 1.54) | | 0.3 | | 0.89 (0.45, 1.76) | | | 0.92 (0.43, 1.96) | | | 0.9 | | | | |
| **24h ratio to urinary creatinine output** | | | | | | | | | | | | | | | | | |
| uAs:Cr µg/mmol | 21.8 (12.6, 37.9) | | 17.3 (11.0, 27.2) | | 0.07 | | 14.9 (8.96, 24.7) | | | 14.0 (8.29, 23.9) | | | 0.7 | | | | |
| uCd:Cr µg/mmol | 3.47 (1.62, 7.41) | | 2.29 (1.05, 4.99) | | 0.002 | | 2.32 (1.04, 5.19) | | | 1.88 (0.83, 4.25) | | | 0.1 | | | | |
| uFe:Cr µg/mmol | 25.9 (9.32, 71.9) | | 13.1 (3.74, 46.1) | | 0.008 | | 13.9 (4.78, 40.2) | | | 11.8 (4.18, 33.5) | | 0.5 | | | | |  |
| uHg:Cr µg/mmol | 0.34 (0.13, 0.87) | | 0.19 (0.08, 0.46) | | ≤0.0001 | | 0.25 (0.11, 0.56) | | | 0.28 (0.12, 0.64) | | | 0.5 | | | | |
| uMn:Cr µg/mmol | 4.64 (1.17, 18.5) | | 2.38 (0.49, 11.3) | | 0.006 | | 2.33 (0.74, 7.35) | | | 2.10 (0.61, 7.27) | | | 0.6 | | | | |
| uMo:Cr µg/mmol | 56.0 (30.5, 103.3) | | 36.6 (20.4, 65.6) | | 0.006 | | 36.9 (21.4, 63.3) | | | 41.9 (20.5, 85.4) | | | 0.4 | | | | |
| uPb:Cr µg/mmol | 2.13 (1.01, 4.52) | | 1.04 (0.60, 1.79) | | ≤0.0001 | | 1.19 (0.62, 2.29) | | | 0.95 (0.39, 2.31) | | | 0.2 | | | | |

The data were positively skewed and are presented as geometric mean (geometric mean-1SD, geometric mean+1SD). Group A: active rickets, Group B: rickets-like bone deformities, As: arsenic, Cd: cadmium, Fe: iron. Hg: mercury, Mn: manganese, Mo: molybdenum, Pb: lead, Cr: Creatinine. Paired t-tests were used to calculate *p*-values for differences between cases and controls in Groups A and B with variables transformed to natural logarithms.
